# Medication administration errors in a Norwegian ambulance service: a quasi-experimental study on the impact of a team training program

**DOI:** 10.1186/s13049-026-01560-1

**Published:** 2026-01-24

**Authors:** Kjetil Myhr, Randi Ballangrud, Jan Porthun, Stephen J M Sollid, Anne Vifladt

**Affiliations:** 1 Department of Health Sciences, Faculty of Medicine and Health Sciences, Norwegian University of Science and Technology, Gjøvik, Norway; 2 Department of Acute Care Medicine, Division Elverum-Hamar, Innlandet Hospital Trust, Hamar, Norway; 3 Department of Prehospital Care, Division Prehospital, Innlandet Hospital Trust, Brumunddal, Norway; 4 Center for Resilience in Healthcare, Faculty of Health Sciences, University of Stavanger, Stavanger, Norway; 5 Department of Research, Innlandet Hospital Trust, Brumunddal, Norway

**Keywords:** Ambulance service, Emergency medical services, Prehospital emergency care, Team training, TeamSTEPPS, Medication administration errors, Medication error, Quality improvement, Intervention, Patient safety

## Abstract

**Background:**

Ambulance professionals operate in dynamic, time-pressured environments where patient safety is paramount, with medication administration errors (MAEs) being a particular concern. While pediatric-focused interventions have addressed dosing errors, few studies have explored strategies to reduce MAEs in prehospital settings. Team Strategies and Tools to Enhance Performance and Patient Safety (TeamSTEPPS) is an evidence-based team training program that has demonstrated positive outcomes in various in-hospital contexts. This study aimed to evaluate the impact of a TeamSTEPPS intervention on MAE frequency in ambulance services.

**Methods:**

This quasi-experimental, pre-post study was conducted within a Norwegian ambulance service across seven ambulance stations split into two groups as part of the TEAM-AMB project. The intervention consisted of a nine-month TeamSTEPPS team training program. Two independent reviewers assessed randomly selected electronic patient journals from pre- and post-intervention periods for MAEs, defined as deviations from the "five rights" of medication administration according to ambulance service protocols. Statistical analysis included descriptive statistics, Chi-square/Fisher's exact tests, Mann–Whitney U tests, and multivariable logistic regression. Cohen's Kappa evaluated interrater reliability.

**Results:**

Overall, 30.6% of ambulance missions contained at least one MAE, with *wrong dose* (17.5%) and *wrong drug* (15.1%) being the most common error subcategories. There was no significant change, combined or for either group, in MAE frequency between pre-intervention (28.9%) and post-intervention (32.2%) periods (*p* = 0.17). *Wrong drug* errors significantly increased from 11.2% to 19.1% post-intervention (*p* < 0.01). The number of different medications administered was the strongest predictor of errors, with each additional medication type increasing error odds by 47% (*p* < 0.01). Patient and mission characteristics showed no association with MAEs in multivariable analysis.

**Conclusions:**

This Norwegian ambulance service study found MAEs in 30.6% of 1,499 missions. The TeamSTEPPS team training intervention did not reduce overall error frequency. The results suggest that team training alone is not sufficient to address the multifaceted causes of MAEs. Future interventions should focus on organizational improvements, particularly enhanced standard operating procedure adherence and electronic documentation systems, to improve accuracy and enable reliable medication error detection.

**Trial registration:**

ClinicalTrials.gov—ID: NCT05244928.

**Supplementary Information:**

The online version contains supplementary material available at 10.1186/s13049-026-01560-1.

## Background

Ambulance services are now an integral component of specialized healthcare, with an expanding scope of practice and heightened expectations for early diagnostics and therapies [1]. Ambulance professionals (APs) work in dynamic teams and complex, time-pressured environments that pose numerous patient safety challenges [2, 3]. In a systematic review on safety in ambulance services, O'Connor et al. reported a wide variability in patient safety incidents, such as medication or diagnostic errors, ranging from 0% to 71% [4]. This substantial variation likely reflects differences in study design, reporting practices, and definitions of incidents across research settings.

Medication errors represent a major patient safety concern worldwide, accounting for 39% of reported adverse events in inpatient settings [5] and significantly contributing to mortality, morbidity, and healthcare costs [6]. The National Coordinating Council for Medication Error Reporting and Prevention (United States) defines medication errors as "any preventable event that may cause or lead to inappropriate medication use or patient harm while the medication is in the control of the healthcare professional, patient, or consumer" [7], a definition endorsed by the World Health Organization [8]. Medication administration errors (MAEs) relate to errors in the preparation and administration of medications [8] and can be categorized by deviations from the widely accepted "five rights" of medication administration: right drug, right dose, right time, right route, and right patient [9]. While the ‘five rights’ provide a fundamental guide to safe medication administration, they do not capture the full complexity of the process, nor does adherence to them guarantee the prevention of MAEs or adverse drug events [10].

Recognizing the burden associated with medication errors, multiple efforts to reduce the frequency of in-hospital medication errors have been undertaken [11]. However, in ambulance services, most interventions have primarily focused on the pediatric population and medication dosing errors [12–14]. Few studies have addressed broader medication safety issues across all age groups. One prospective observational study targeting all age groups implemented a medication administration cross-check procedure that resulted in a 49% relative risk reduction of self-reported medication errors [15]. Additionally, a structured inspection study of medication storage and handling practices in an ambulance service in the United States identified 46 potential safety issues, 16 of which were classified as high-risk, such as three different methods of carrying adrenaline [16].

Given the limited evidence for interventions targeting MAEs in ambulance services, a broader team-based approach to patient safety warrants examination. As highlighted in a scoping review by Fisher et al., there is a need to prioritize patient safety in ambulance services, even when operational pressures and public demands are high [17]. Team Strategies and Tools to Enhance Performance and Patient Safety (TeamSTEPPS) is an evidence-based team training program created to improve patient safety and has been implemented in numerous in-hospital and primary care settings worldwide [18, 19]. TeamSTEPPS focuses on four key skills that contribute to team effectiveness: communication, team leadership, situation monitoring, and mutual support [18]. Several studies have demonstrated the benefits of team training and proficient teamwork in emergency medicine and ambulance services, including improved structure and quality of communication [2], enhanced care of simulated critically ill and injured patients [20, 21], and reduced frequency of medical errors [22, 23].

Despite these promising findings, the specific impact of team training on MAEs is not well known. Therefore, this study aims to evaluate the impact of a team training program — TeamSTEPPS — on the frequency of MAEs in a Norwegian ambulance service.

## Methods

### Design

This study employed a quasi-experimental, pre-post design and is part of the TEAM-AMB project [24]. The Transparent Reporting of Evaluations with Nonrandomized Designs (TREND) statement was used to guide the reporting of the study [25]; see additional file 1.

### Research paradigm and theoretical framework

The study was conducted within a postpositivist research paradigm, which acknowledges an objective reality that can only be imperfectly understood due to inherent limitations in human observation and measurement [26]. The TeamSTEPPS intervention is based on the theoretical framework outlined in the 'Big Five' model of teamwork [27], while the TEAM-AMB project employs The Systems Engineering Initiative for Patient Safety (SEIPS) model as a theoretical framework to understand the complexity of medication administration in ambulance services [28].

### Setting

The ambulance service in Norway is an integral part of the public healthcare system and is organized into 18 separate services under locally managed, state-owned hospital trusts. This study was conducted within one of these ambulance services, involving ambulance professionals (APs) from seven ambulance stations that were chosen by the hospital trust and collectively serve a population of approximately 150,000 residents. To facilitate the implementation of the team training program, the seven stations were divided into two groups, each covering both urban and rural areas and handling around 10,000 missions per year. All APs are certified healthcare professionals under Norwegian law, qualified either as emergency medical technicians (EMTs), paramedics, or registered nurses with additional certification to work in ambulance services [29]. All APs who administer medications have undergone theoretical and practical training, with local guidelines and standard operating procedures dictating the scope of medication administration privileges that each individual AP has. Deviations from these protocols require documentation and if feasible, should be discussed with a consulting physician prior to administration. APs document their care in the electronic patient journal (EPJ) during or after patient care using a touch-screen tablet with a separate keyboard. During the study period, the ambulance service had 29 different medications available, with some medications available in multiple formulations and concentrations.

### Intervention

In this study, the intervention involved the implementation of the TeamSTEPPS team training program. A selected group of APs received master training to serve as TeamSTEPPS instructors and formed Change Teams together with the ambulance station leaders and the first author (KM). The two Change Teams, one for each group, was responsible for planning the team training and selecting appropriate TeamSTEPPS tools and strategies to fit the needs of the ambulance service. The content of the intervention was similar for both groups, and the order in which they started the team training was randomized using a computer-generated sequence.

The intervention started with a full mandatory introduction day with lectures, group exercises and discussions. All APs who worked regular shifts at the seven ambulance stations were invited to participate. For each of the following four months, the focus was on improving a single key skill (communication, team leadership, situation monitoring or mutual support) and the implementation of selected strategies and tools (such as giving feedback or ISBAR). Particularly relevant to this study was the focus on situation monitoring during medication administration. The APs were encouraged to cross-check medications in a way inspired by the *Medication Administration Cross Check* (MACC) procedure [15] (see additional file 2) as well as to label all syringes and report adverse and potentially adverse events related to medication administration.

Following a two-month break after the initial intervention period, a three-month sustainment phase was conducted. During this phase, APs were encouraged to provide verbal feedback to their colleagues on the use and development of teamwork skills. Concurrently, members of the Change Team worked to integrate TeamSTEPPS principles into the ambulance service's regular operations through established educational initiatives and simulation training. A more detailed description of the intervention can be found in additional file 3.

### Sample and data collection

Due to the wide range of MAE frequencies reported in previous studies [4], we chose to review an initial sample of 500 EPJs from the two groups that would take part in the intervention. Following a pre-planned interim analysis of these data, we determined that additional EPJs were required to achieve adequate statistical power, bringing our total pre-intervention sample to 750 EPJs.

Using results from the pre-intervention sample, we calculated the required post-intervention sample size. A clinically relevant reduction in MAEs was set at 10% by consensus within the research group. To detect a 10% difference with 85% power and a Type I error rate of 0.05, the post-intervention sample size was calculated to include 748 EPJs after accounting for dropouts. Sample size calculations were performed using PASS 2022 (version 22.0.3, NCSS Statistical Software, Kaysville, UT, USA).

EPJs documenting the administration of at least one medication were included. EPJs without sufficient information were excluded at the discretion of the reviewers. Examples of exclusions included missing essential clinical data (such as patient vital signs, chief complaint, or medical history) or incomplete medication information (including medication names, dosages, administration routes, or timing).

**Fig. 1:**
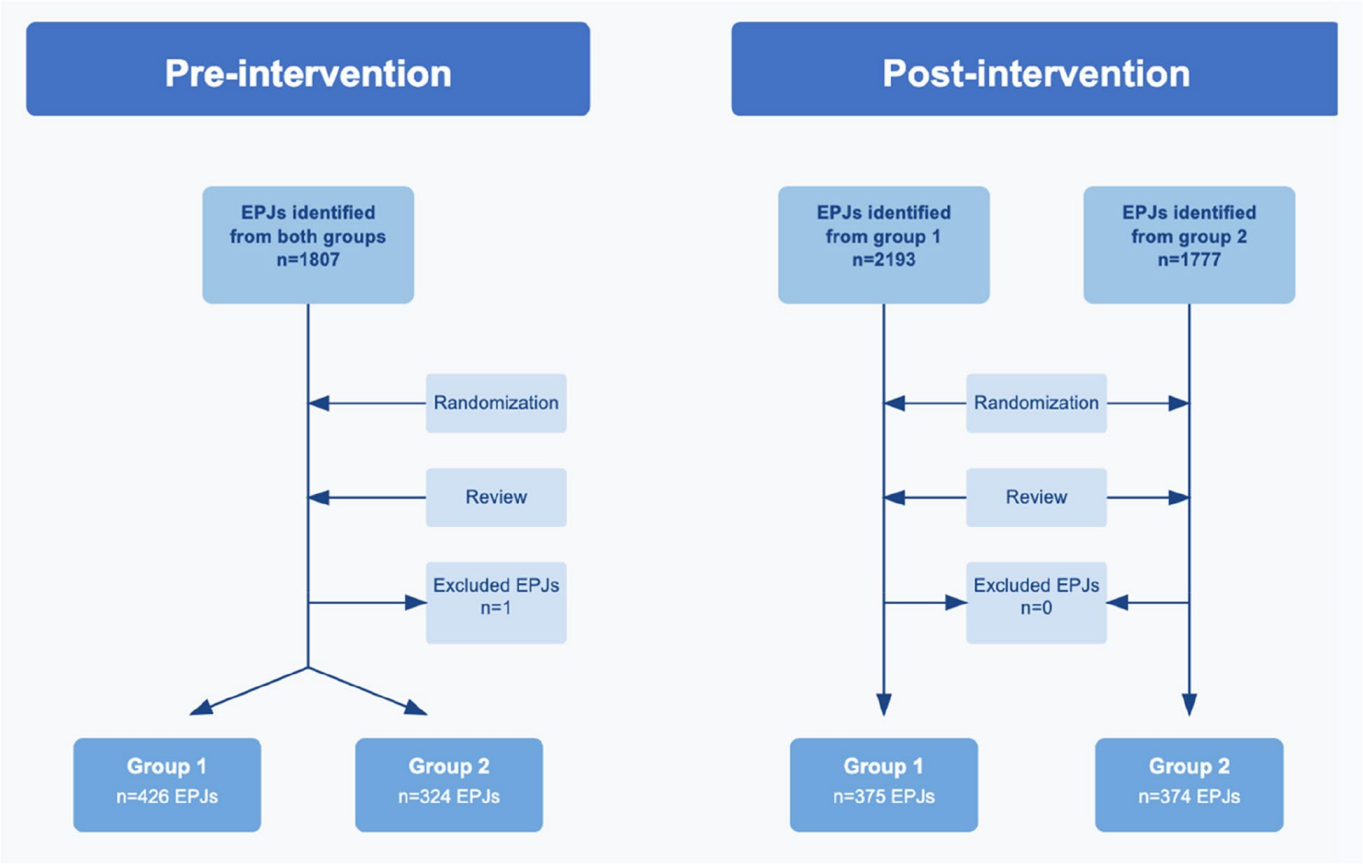
Electronic patient journal flowchart. In the pre-intervention period, electronic patient journals (EPJs) from both groups were pooled before randomization and review. In the post-intervention period, randomization and review were performed within each group separately

**Fig. 2:**
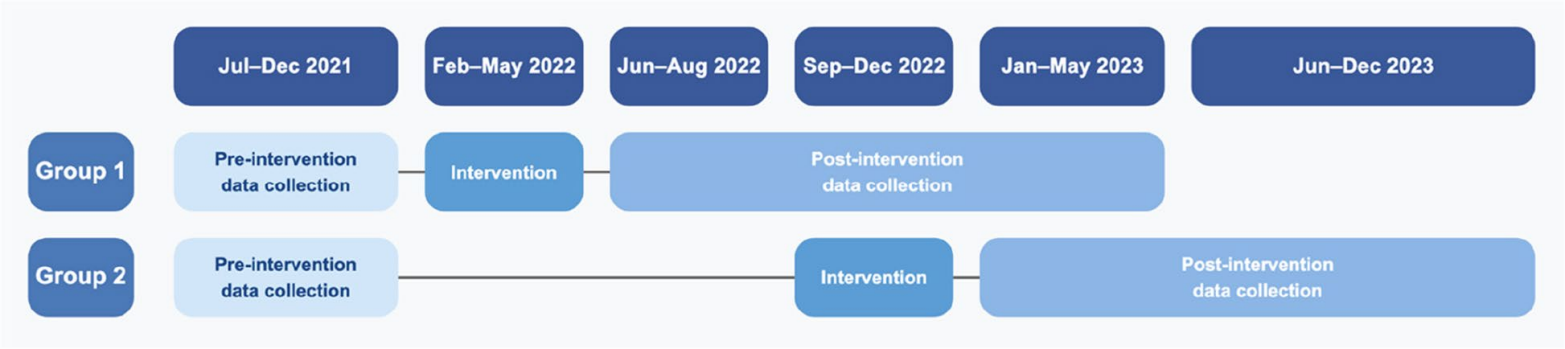
Timeline of data collection and intervention. The sustainment phase of team training occurred during the post-intervention data collection period

All included EPJs were assigned unique IDs and randomized using computer-generated sequences. The flowchart in Fig. 1 illustrates the process of EPJ selection and randomization, while the timeline in Fig. 2 shows the relationship between the intervention and data collection for the two groups.

### Outcomes

The primary outcome was the change in frequency of EPJs containing at least one MAE between pre- and post-intervention periods. Secondary outcomes included changes in the frequency of MAE subcategories and associations between MAEs and independent variables. Independent variables included the intervention, patient sex, patient age categories (0–5, 6–11, 12–17, 18–39, 40–69, or > 69 years), mission start day (Monday-Friday vs. Saturday-Sunday), mission start time (08:00–19:59 vs. 20:00–07:59), mission duration, mission triage (red, orange, yellow, or green), medication doses administered, and number of different medications administered.

### Record review process

Two reviewers, an anesthesiologist with prehospital experience (KM) and a paramedic/prehospital nurse (EG pre-intervention and BKT post-intervention), conducted a non-blinded separate review of EPJs according to the review protocol, see additional file 4. Pilot reviews of 10 EPJs from outside the study sample were conducted to familiarize the reviewers (KM/EG/BKT/SS) with the process and facilitate discussions on challenging cases. MAEs were defined as deviations from the 5Rs [9], according to the medication protocols of the ambulance service. When the reviewers identified an MAE, the type of error was classified according to the categories: *wrong drug, wrong dose, wrong time, wrong route* or *other.* Medications that should be dosed by weight but where the patient’s weight was not reported were categorized separately as a *weight-dose omission*, but not an MAE. Intravenous fluids and oxygen were not assessed as medications in this study. Upon completion of the independent reviews, all EPJs where the two reviewers disagreed were reevaluated. For those EPJs where consensus could not be reached, a third expert reviewer (SS) was consulted to finalize a decision. The record review process took place from November 2022 to January 2023 for the pre-intervention data, and in January 2024 for the post-intervention data.

### Statistical analysis

Descriptive statistics were used to summarize sample characteristics and MAEs. Means and standard deviations (SD) were reported for normally distributed variables, while we used medians and interquartile ranges (IQR) for non-normally distributed variables. Normality was assessed through visual inspection of histograms and Q-Q plots. Cohen’s Kappa (κ) was used to assess interrater reliability between the two primary reviewers. The following description of agreement was used: < 0.00 poor, 0.00–0.20 slight, 0.21–0.40 fair, 0.41–0.60 moderate, 0.61–0.80 substantial, and 0.81–1.00 almost perfect [30].

To compare groups and assess associations between MAEs and independent variables, Chi-square tests (or Fisher’s exact tests when expected cell counts were < 5) were used for nominal variables. The Mann–Whitney *U* test was used for ordinal and continuous variables that were not normally distributed.

Before conducting multivariable analyses, we assessed collinearity between independent variables using the Variance Inflation Factor (VIF), where values above 5 indicated potential concerns. Univariable logistic regression analyses were first performed to examine crude associations between each independent variable and MAE frequency. Variables were then included in a multivariable logistic regression model to identify independent predictors while adjusting for all other variables. The model included the intervention as the primary predictor and was adjusted for group, sex, age categories, mission characteristics (weekday/weekend, day/night, duration), triage category, medication doses administered, and number of different medications administered. All predefined independent variables were included in the multivariable model regardless of univariate significance to control for potential confounding. Missing data were handled using complete case analysis. Outliers were assessed using standardized residuals with a threshold of ± 2.5 SD. To evaluate whether the effect of the intervention differed between the two groups, we tested for the interaction term [Group × Intervention].

*P*-values < 0.05 were considered statistically significant. All analyses were performed using IBM SPSS Statistics for Windows, Version 30.0 (IBM Corp., Armonk, NY, USA).

### Language editing

The manuscript was copy edited and its readability enhanced using Microsoft 365 Copilot.

## Results

### Sample characteristics

A total of 1,499 EPJs were included in the final analysis, with 750 and 749 EPJs from the pre- and post-intervention periods, respectively. Patient demographic characteristics, stratified by sex and age categories, are presented in Table 1. In total, there was a slight majority (52.6%) of male patients. Nearly half of the population (48.0%) was elderly (> 69 years), while 3.6% were pediatric patients (< 18 years old).

**Table 1:** Patient characteristics

	**Pre-intervention**			**Post-intervention**			**Total**
	**Both groups**	**Group 1**	**Group 2**	**Both groups**	**Group 1**	**Group 2**	
**Number of missions/EPJs, n**	750	426	324	749	375	374	1499
**Sex**							
Female, n (%)	347 (46.3)	199 (46.7)	148 (45.7)	364 (48.6)	188 (50.1)	176 (47.1)	711 (47.4)
Male, n (%)	403 (53.7)	227 (53.3)	176 (54.3)	385 (51.4)	187 (49.9)	198 (52.9)	788 (52.6)
**Age**							
0–5 years, n (%)	7 (0.9)	4 (0.9)	3 (0.9)	9 (1.2)	7 (1.9)	2 (0.5)	16 (1.1)
6–11 years, n (%)	2 (0.3)	0 (0.0)	2 (0.6)	11 (1.5)	5 (1.3)	6 (1.6)	13 (0.9)
12–17 years, n (%)	8 (1.1)	3 (0.7)	5 (1.5)	16 (2.1)	3 (0.8)	13 (3.5)*	24 (1.6)
18–39 years, n (%)	80 (10.7)	52 (12.2)	28 (8.6)	116 (15.5)	50 (13.3)	66 (17.6)	196 (13.1)
40–69 years, n (%)	272 (36.3)	148 (34.7)	124 (38.3)	259 (34.6)	124 (33.1)	135 (36.1)	531 (35.4)
> 69 years, n (%)	381 (50.8)	219 (51.4)	162 (50.0)	338 (45.1)	186 (49.6)	152 (40.6)*	719 (48.0)

Chi-square tests were used to compare groups with cell counts > 5, and Fisher’s exact tests for cell counts ≤ 5

*EPJs* Electronic patient journals

^*^ marks significant differences (*p* < 0.05) between groups

Descriptive statistics for mission characteristics are presented in Table 2. Missions were similarly distributed across days of the week, with most (64.3%) starting during daytime hours (08:00–19:59). Data on mission duration were available for 1,350 EPJs (90.1%), the remaining 149 EPJs (9.9%) were non-conveyance missions where the patient was treated and left on scene. Patient triage data, a surrogate for mission acuity, were available for 1,476 EPJs (98.5%). The highest acuity missions were classified as red and represented 21.2% of the sample, while green missions (11.2%) represented the lowest acuity. About half (51.2%) of the patients received only one type of medication, while 2.8% received four or more different types of medications. In total, 3,176 individual medication doses were administered across the sample. The most frequently administered medication was morphine (32.4%), followed by paracetamol (11.5%) and nitroglycerin (10.5%).

**Table 2:** Mission characteristics

	**Pre-intervention**			**Post-intervention**			**Total**
	**Both groups**	**Group 1**	**Group 2**	**Both groups**	**Group 1**	**Group 2**	
**Number of missions/EPJs, *n***	750	426	324	749	375	374	1499
**Mission start—weekday/weekend**							
Monday-Friday, *n* (%)	555 (74.0)	321 (75.4)	234 (72.2)	535 (71.4)	281 (74.9)	254 (67.9)*	1090 (72.7)
Saturday-Sunday, *n* (%)	195 (26.0)	105 (24.6)	90 (27.8)	214 (28.6)	94 (25.1)	120 (32.1)*	409 (27.3)
**Mission start—day/night**							
Day 08:00–19:59, *n* (%)	465 (62.0)	262 (61.5)	203 (62.7)	499 (66.6)	231 (61.6)	268 (71.7)*	964 (64.3)
Night 20:00–07:59, *n* (%)	285 (38.0)	164 (38.5)	121 (37.3)	250 (33.4)	144 (38.4)	106 (28.3)*	535 (35.7)
**Mission duration**							
Minutes, median (IQR)	51 (39–71)	47 (37–62)	60 (42–82)*	55 (40–74)	51 (38–64)	60 (41–89)*	53 (39–72)
Missing, *n* (%)	89 (11.9)	58 (13.6)	31 (9.6)	60 (8.0)	29 (7.7)	31 (8.3)	149 (9.9)
**Triage**							
Red, *n* (%)	159 (21.5)	98 (23.2)	61 (19.4)	154 (20.9)	85 (22.9)	69 (18.8)	313 (21.2)
Orange,* n* (%)	240 (32.5)	129 (30.5)	111 (35.2)	248 (33.6)	114 (30.8)	134 (36.4)	488 (33.1)
Yellow, *n *(%)	254 (34.4)	145 (34.3)	109 (33.6)	256 (34.7)	136 (36.8)	120 (32.6)	510 (34.6)
Green, *n *(%)	85 (11.5)	51 (12.1)	34 (10.8)	80 (10.8)	35 (9.5)	45 (12.2)	165 (11.2)
Missing,* n *(%)	12 (1.6)	3 (0.7)	9 (2.8)	11 (1.5)	5 (1.3)	6 (1.6)	23 (1.5)
**Medication doses administered**							
1, *n* (%)	316 (42.1)	165 (38.7)	151 (46.6)*	321 (42.9)	149 (39.7)	172 (46.0)	637 (42.5)
2–3, *n* (%)	332 (44.3)	205 (48.1)	127 (39.2)*	335 (44.7)	186 (49.6)	149 (39.8)*	667 (44.5)
> 3, *n* (%)	102 (13.6)	56 (13.1)	46 (14.2)	93 (12.4)	40 (10.7)	53 (14.2)	195 (13.0)
**Number of different medications administered**							
Median (IQR)	1 (1–2)	2 (1–2)	1 (1–2)*	1 (1–2)	2 (1–2)	1 (1–2)	1 (1–2)

Chi-square tests were used to compare groups with normal distribution, and the Mann–Whitney U test for continuous variables that were not normally distributed

*EPJs* Electronic patient journals, *IQR* Interquartile range

^*^ marks significant differences (*p* < 0.05) between Group 1 and 2

Combined, Group 1 had higher patient age categories than Group 2 (mean ranks 771.15 vs 725.73, *p* = 0.03). Other significant differences between groups are indicated in Tables 1 and 2. Within-group analysis revealed minimal changes in Group 1 pre- to post-intervention, with only a significant shift toward more patients aged 6–11 years post-intervention (*p* = 0.02). In contrast, Group 2 demonstrated greater changes from pre- to post-intervention: more patients aged 18–39 years (*p* < 0.01), fewer patients aged > 69 years (*p* = 0.01), and an increase in daytime mission starts (*p* = 0.01).

### Medication administration errors and impact of the team training intervention

Regarding the primary outcome, there was no significant change in MAE frequency when comparing pre-intervention (28.9%) to post-intervention periods (32.2%) (*p* = 0.17). Overall, 30.6% of EPJs contained at least one MAE, see Table 3.

**Table 3:** Medication administration errors pre- and post-intervention

	**Both groups**				**Group 1**			**Group 2**		
	**Pre-intervention**	**Post-intervention**	***p*-value**	**Total**	**Pre-intervention**	**Post-intervention**	***p*-value**	**Pre-intervention**	**Post-intervention**	***p*-value**
**Number of missions/EPJs,** *n*	750	749		1499	426	375		324	374	
**MAEs,** *n* (%)	217 (28.9)	241 (32.2)	0.17	458 (30.6)	126 (29.6)	125 (33.3)	0.25	91 (28.1)	116 (31.0)	0.40
**Subcategory MAEs**										
Wrong drug, *n *(%)	84 (11.2)	143 (19.1)	** < 0.01**	227 (15.1)	46 (10.8)	66 (17.6)	** < 0.01**	38 (11.7)	77 (20.6)	** < 0.01**
Wrong dose, *n* (%)	142 (18.9)	120 (16.0)	0.14	262 (17.5)	85 (20.0)	70 (18.7)	0.65	57 (17.6)	50 (13.4)	0.12
Wrong time, *n* (%)	6 (0.8)	5 (0.7)	1.00	11 (0.7)	1 (0.2)	2 (0.5)	0.60	5 (1.5)	3 (0.8)	0.48
Wrong route, *n* (%)	6 (0.8)	9 (1.2)	0.43	15 (1.0)	2 (0.5)	4 (1.1)	0.43	4 (1.2)	5 (1.3)	1.00
Other, *n* (%)	0 (0.0)	2 (0.3)	0.25	2 (0.1)	0 (0.0)	1 (0.3)	0.47	0 (0.0)	1 (0.3)	1.00

Chi-square tests were used to compare groups with cell counts > 5, and Fisher’s exact tests for cell counts ≤ 5; Each mission/EPJ was classified as having an MAE or not; Missions could have one or more subcategories of MAEs

*EPJs* Electronic patient journals

Significant differences (*p* < 0.05) are marked in bold

For the secondary outcomes, the only significant change in subcategory MAEs was the increased frequency of *wrong drug* errors from 11.2% pre-intervention to 19.1% post-intervention (*p* < 0.01). The most common subcategories of MAEs were *wrong dose* (17.5%) and *wrong drug* (15.1%), while *wrong time, wrong route,* and *other* occurred infrequently (< 2.0% combined).

Administration of ondansetron, an antiemetic, accounted for 3.6% (3/84) of *wrong drug* MAEs pre-intervention and 18.2% (26/143) post-intervention. Even after excluding ondansetron-related errors, the increase in *wrong drug* MAEs remained statistically significant (*p* < 0.01). There was a 29.3% relative increase in the number of patients receiving ondansetron between the two periods: 79/750 pre-intervention and 102/749 post-intervention (*p* = 0.07).

In 26.8% of all EPJs, we recorded a *weight-dose omission* without an MAE, meaning that a medication that should be dosed by weight was administered but no weight was documented in the EPJ. Combined for Group 1 and 2, w*eight-dose omissions* decreased from 29.2% pre- to 24.3% post-intervention (*p* = 0.03).

Univariable and multivariable logistic regression analyses were performed to identify factors associated with MAEs (Table 4 and additional file 5). The final model was statistically significant (χ^2^ = 45.36, df = 17, *p* < 0.01), explaining 4.7% of variance in MAE frequency (Nagelkerke R^2^). The Hosmer–Lemeshow test indicated good model fit (*p* = 0.81). The intervention did not significantly impact the frequency of MAEs (adjusted OR = 1.17, 95% CI: 0.92–1.48, *p* = 0.21). The number of different medications administered was the only factor significantly associated with MAEs. Each additional medication type increased the MAE odds by 47% (adjusted OR = 1.47, 95% CI: 1.18–1.82, *p* < 0.01). In univariable analysis, administering > 3 medication doses was associated with MAEs (OR = 2.19, 95% CI: 1.57–3.05, *p* < 0.01), but this association was attenuated after adjustment (adjusted OR = 1.14, *p* = 0.65).

**Table 4:** Univariable and multivariable logistic regression analysis of medication administration errors

	**Univariable model**			**Multivariable model**		
**Variable**	**OR**	**95% CI**	***p*-value**	**Adjusted OR**	**95% CI**	***p*-value**
**Intervention**	1.17	0.94–1.45	0.17	1.17	0.92–1.48	0.21
**Group**	1.08	0.87–1.35	0.48	1.06	0.83–1.36	0.63
**Sex**	1.08	0.86–1.34	0.52	1.10	0.86–1.39	0.46
**Age** (ref: > 69 years)			0.82			0.68
0–5 years	0.95	0.33–2.78	0.93	1.76	0.55–5.66	0.35
6–11 years	0.93	0.28–3.06	0.91	1.13	0.33–3.85	0.85
12–17 years	1.05	0.44–2.49	0.91	0.80	0.30–2.11	0.65
18–39 years	0.84	0.59–1.19	0.32	0.88	0.60–1.30	0.52
40–69 years	0.85	0.67–1.09	0.19	0.84	0.64–1.09	0.19
**Mission start – weekday/weekend**	1.05	0.82–1.34	0.71	1.14	0.87–1.50	0.35
**Mission start – day/night**	0.88	0.70–1.10	0.26	0.87	0.68–1.12	0.28
**Mission duration**	1.00	1.00–1.01	0.28	1.00	1.00–1.01	0.72
**Triage** (ref: Green)			0.98			0.78
Red	1.02	0.68–1.53	0.93	0.95	0.59–1.53	0.84
Orange	1.00	0.68–1.47	0.99	1.03	0.66–1.60	0.91
Yellow	0.96	0.66–1.40	0.83	1.13	0.73–1.75	0.60
**Medication doses administered** (ref: 1 dose)			< 0.01			0.53
2–3 Doses	1.21	0.95–1.54	0.12	0.93	0.66–1.30	0.66
4 + Doses	2.19	1.57–3.05	< 0.01	1.14	0.65–2.00	0.65
**Number of different medications administered**	1.42	1.25–1.61	< 0.01	1.47	1.18–1.82	< 0.01

Missions included in analysis, *n* = 1,333 (88.9% of 1,499 missions); Overall classification accuracy: 69.1%

*OR* odds ratio, *CI* confidence interval

For each MAE subcategory, separate multivariable logistic regression analyses were conducted using the same predictor variables as the primary regression analysis. Due to small sample sizes, only *wrong drug* and *wrong dose* provided reliable results. Overall, there was an increase in *wrong drug* MAEs post-intervention (adjusted OR = 2.02, 95% CI: 1.47–2.76, *p* < 0.01). Significant predictors included the number of different medications administered (adjusted OR = 1.45, 95% CI: 1.11–1.89, *p* = 0.01) and missions starting at night (adjusted OR = 1.39, 95% CI: 1.01–1.90, *p* = 0.04).

No significant change in *wrong dose MAEs* was found post-intervention (adjusted OR = 0.83, 95% CI: 0.62–1.10, *p* = 0.19). Males were associated with lower odds of *wrong dose* MAEs (adjusted OR = 0.67, 95% CI: 0.50–0.90, *p* = 0.01), while the number of different medications administered remained a significant predictor (adjusted OR = 1.31, 95% CI: 1.03–1.68, *p* = 0.03).

### Reviewer agreement

Interrater reliability was κ 0.81 (95% CI: 0.77–0.85) in the pre-intervention period and κ 0.77 (95% CI: 0.72–0.81) in the post-intervention period indicating substantial to almost perfect agreement. To reach consensus, third reviewer consultation (SS) was required for seven EPJs pre-intervention and five post-intervention.

## Discussion

This quasi-experimental study aimed to evaluate the impact of a team training program — TeamSTEPPS — on the frequency of MAEs in a Norwegian ambulance service. The intervention did not lead to a reduction in MAE frequency, as rates remained statistically unchanged between the pre- and post-intervention periods.

### Medication administration errors and associated factors

Overall, nearly one-third of ambulance missions in this study had at least one MAE. These results are similar to other ambulance service record reviews, particularly the study by Ramadanov et al. [31]. Record reviews generally report higher frequency of MAEs than alternative detection methods. For instance, self-reported errors by APs are notoriously under-reported [32], with rates as low as 0.1% [15], while trigger tool studies have identified medication-related adverse events at frequencies of 0.5% [33] and 4.3% [34].

This study found no significant difference in MAE frequency across age groups, aligning with two previous studies [34, 35]. However, other research has yielded conflicting results, with one study finding more MAEs specifically in pediatric patients [36] and another in both pediatric and elderly populations [31]. The limited number of pediatric patients in this study, however, restricts our ability to detect potential differences in MAE related to age groups.

Examining timing as a factor, there was no increased frequency of MAEs during nighttime hours in this study. This is consistent with a mixed ambulance and emergency department study [35], but in contrast to the research from Ramadanov et al. [31] that reported higher nighttime error rates. These discrepancies may stem from differences in how nighttime was defined: the Ramadanov study defined it as 03:00–06:00, while this study used a broader period from 20:00–07:59. Including evening hours, when alertness levels may differ from those in the early morning, could have diluted any potential difference.

No association was detected between mission triage level, a surrogate for patient condition severity, and MAEs. This finding is similar to that by Lifschitz et al. [35]. However, previous research has suggested that mission severity is linked to adverse events [33, 37]. One possible explanation is that the most severe missions in this study were more likely to involve physicians in addition to APs. Because physicians are not bound by the ambulance service’s standard operating procedures like APs, deviations from medication protocols were not classified as MAEs when a physician was responsible for the patient’s care. This may have led to underestimation of MAEs in higher triage missions.

The number of different medications administered emerged as the only predictor consistently associated with increased MAE risk in our study, whereas the total number of doses administered was not an independent predictor in the multivariable analysis. This distinction likely reflects that repeated doses primarily involved titrated analgesics with lower error potential, while administering multiple different medications increases complexity and error opportunities, a relationship supported by existing literature [38]. These findings raise important questions about which medications should be carried by ambulance services, given that expanding scopes of practice introduce both benefits and inherent risks [39].

The multivariable logistic regression model explained only 4.7% of the variance in MAE frequency, suggesting that unmeasured factors beyond routine documentation account for most errors. According to human factors principles, healthcare errors arise from complex interactions among multiple work system components including people (e.g., AP experience and certification level), environments (physical conditions like lighting and space), tools (standard operating procedures), and tasks [40]. The low explained variance suggests that MAEs likely result from dynamic interactions among these factors rather than simple, directly measurable variables.

### Impact of the TeamSTEPPS team training program

The intervention did not lead to a reduction in MAE frequency; instead, a surprising increase in *wrong drug* errors was observed in the post-intervention period. Several factors may help explain these findings:

First, team training alone may not adequately address all the main causes of MAEs. In the systematic review by Walker et al. [38], seven factors influencing MAEs in ambulance services were identified through qualitative analysis. Team training is likely to influence only three of these: communication, procedural, and cognitive factors. While organizational and patient-related factors may be indirectly affected, equipment- and environment-related factors are unlikely to be affected.

One key example of this is adherence to standard operating procedures, as medications administered outside of these protocols were classified as MAEs. In a systematic review by Ebben et al. [41], prehospital adherence rates ranged from 7.8% to 95.0%, with the lowest compliance in treatments for myocardial infarction and cardiac arrest. This suggests that interventions which do not address organizational causes of non-compliance—such as ambiguous medication protocols, or procedures that are systematically ignored because they are considered inappropriate or impractical—are less likely to reduce MAE frequency.

Second, lack of AP compliance with the intervention and personnel turnover may have limited effectiveness of the team training. Adherence to the components of the intervention that specifically targeted medication administration, such as situation monitoring and cross-checking, was lower compared to other parts of the team training [2], reducing the likelihood of impact. In addition, not all APs completed the full team training program. One year after the intervention ended, approximately 25% of APs were no longer employed at the same ambulance service (ambulance station leaders, personal communication, January 31, 2024). It is possible that the team training might have yielded positive effects if implemented in a different context or with improved fidelity [42].

Third, the failure of team training to reduce MAEs may be explained by changes in documentation practices and the nature of identified MAEs. The intervention may have fostered an improved safety climate, leading to more thorough documentation and detection of previously undocumented MAEs [43], consistent with the "reporting paradox" where increased error reporting indicates healthier safety climate rather than deteriorating practice [44]. This hypothesis is supported by the significant reduction in weight-dose omissions, highlighting how the APs more often documented the patient's weight post-intervention.

However, the record review process also classified obvious documentation errors as MAEs. These included cases where multiple titrated doses were incorrectly summed as single enormous doses, or where medication formulations or doses not carried by ambulances were recorded—errors that almost certainly never occurred in practice. While poor documentation has been linked to adverse events [33], many identified MAEs likely represent documentation errors rather than actual errors with potential for patient harm. This aligns with the work by England et al. [45], who found that half of prehospital MAEs were documentation errors.

Considering the early, and possibly temporary, changes that could have occurred after the intervention, such as more accurate documentation by APs, coupled with the fact that it takes time for organizational changes to settle after most health care interventions [46], extended follow-up measurements of MAEs could provide a clearer picture of the team training program’s long-term effects.

Further, while the identification of MAEs was based on objective criteria, it was still reliant on subjective evaluation, and it is possible that the review team post-intervention was stricter in their assessments. This possibility is supported by the five-fold increase in wrong drug MAEs involving the antiemetic ondansetron, which far exceeded the rise in ondansetron administrations post-intervention. This disproportionate rise suggests increased detection sensitivity among the reviewers for this particular medication, rather than an actual increase in *wrong drug* MAEs. Although the review teams demonstrated substantial to almost perfect interrater agreement within their respective periods [30], this does not preclude systematic differences in interpretation between the pre- and post-intervention review processes.

Finally, several methodological considerations may explain why the intervention failed to reduce MAE frequency and why wrong drug errors increased. These include unmeasured confounders, temporal effects (such as changes in systems, policy, or staffing) and false positive findings due to multiple statistical comparisons. Given these limitations, the results should be interpreted with caution, as generalizability to other ambulance services will depend on differences in implementation fidelity, organizational safety culture, and existing teamwork practices.

### Implications and future directions

The finding that nearly one-third of ambulance missions involved MAEs reveals an important patient safety issue. Our findings point to important areas that warrant further attention.

First, we need systems that allow more reliable detection of MAEs, rather than errors attributable to documentation. EPJ systems should be developed to improve documentation accuracy by preventing the entry of unavailable medications or doses, and they should be better aligned with AP workflow.

Future research should address the limited knowledge on how and why MAEs with potential for harm occur, providing insights to guide tailored interventions. Ultimately, these interventions should adopt multifaceted approaches that combine individual and system-level improvements—such as team training to improve patient safety culture and increase adherence to clear, concise standard operating procedures.

### Limitations

This study has important methodological limitations. The most significant limitation involves the study design: we originally planned a stepped-wedge cluster randomized trial, which would have provided stronger evidence for causality and intervention effectiveness [47], but shifted to a quasi-experimental design with no designated control group due to implementation challenges. Key issues included having only two study groups, a high risk of contamination between groups (control participants potentially exposed to intervention components), and the failure to collect data across both groups until the end of the study period (see Fig. 2). The staggered implementation of the intervention between the two groups could have introduced temporal confounding, as external factors affecting MAEs may have varied between implementation periods [48].

Another limitation concerns the review process. While the primary author (KM) and consulting expert (SS) were part of both review teams, there was a different second reviewer post-intervention, which together with the lack of blinding for the intervention could have introduced detection bias in the form of systematic differences in MAE identification.

Additionally, the randomization process for selecting EPJs, as shown in Fig. 1, differed slightly between the pre- and post-intervention phases. In the pre-intervention phase, randomization was performed on the entire sample, whereas in the post-intervention phase it occurred at the individual group level. This resulted in a greater number of EPJs from Group 1 in the pre-intervention data than in the post-intervention data. However, because the selection remained random, we consider it unlikely that this difference substantially affected the study results.

The study is further limited as we did not assess for errors of omission, which is one of the most common wrong drug MAEs [34]. Nor did we quantify the number of MAEs per EPJ, assess their severity, or identify any resulting adverse events.

Finally, while the study had minimal missing data, these data were not missing at random as the majority represented non-conveyance missions. Consequently, the multivariable logistic regression analysis was conducted without these cases, introducing a minor selection bias.

## Conclusions

This quasi-experimental study in a Norwegian ambulance service found that 30.6% of 1,499 ambulance missions had at least one medication administration error (MAE). While the overall MAE frequency did not change after implementation of a TeamSTEPPS team training program, the rate of *wrong drug* errors unexpectedly increased. These findings highlight the complexity of medication safety in ambulance services and suggest that team training alone is insufficient to address the multifaceted causes of MAEs. Future interventions should focus on system-level improvements with targeted efforts to increase standard operating procedure adherence and improve electronic documentation systems, facilitating accurate documentation by ambulance professionals and enabling more reliable detection and measurement of MAEs.

## Supplementary Information

Additional file 1

Additional file 2

Additional file 3

Additional file 4

Additional file 5

## Data Availability

The data collected and analyzed in this study are not available to the public but can be obtained from the corresponding author upon reasonable request.

## Abbreviations

AP: Ambulance Professional
EPJ: Electronic Patient Journal
MACC: Medication Administration Cross Check
MAE: Medication Administration Error
TeamSTEPPS: Team Strategies and Tools to Enhance Performance and Patient Safety
