# Supplementary material for: Medication administration errors in a Norwegian ambulance service: a quasi-experimental study on the impact of a team training program
Source: Scand J Trauma Resusc Emerg Med. 2026 Jan 24;34:41. doi: 10.1186/s13049-026-01560-1 (PMC12911085; doi:10.1186/s13049-026-01560-1)
Supplement: Supplementary file 2 — Additional file 2 [file 13049_2026_1560_MOESM2_ESM.pptx]

## Slide 1
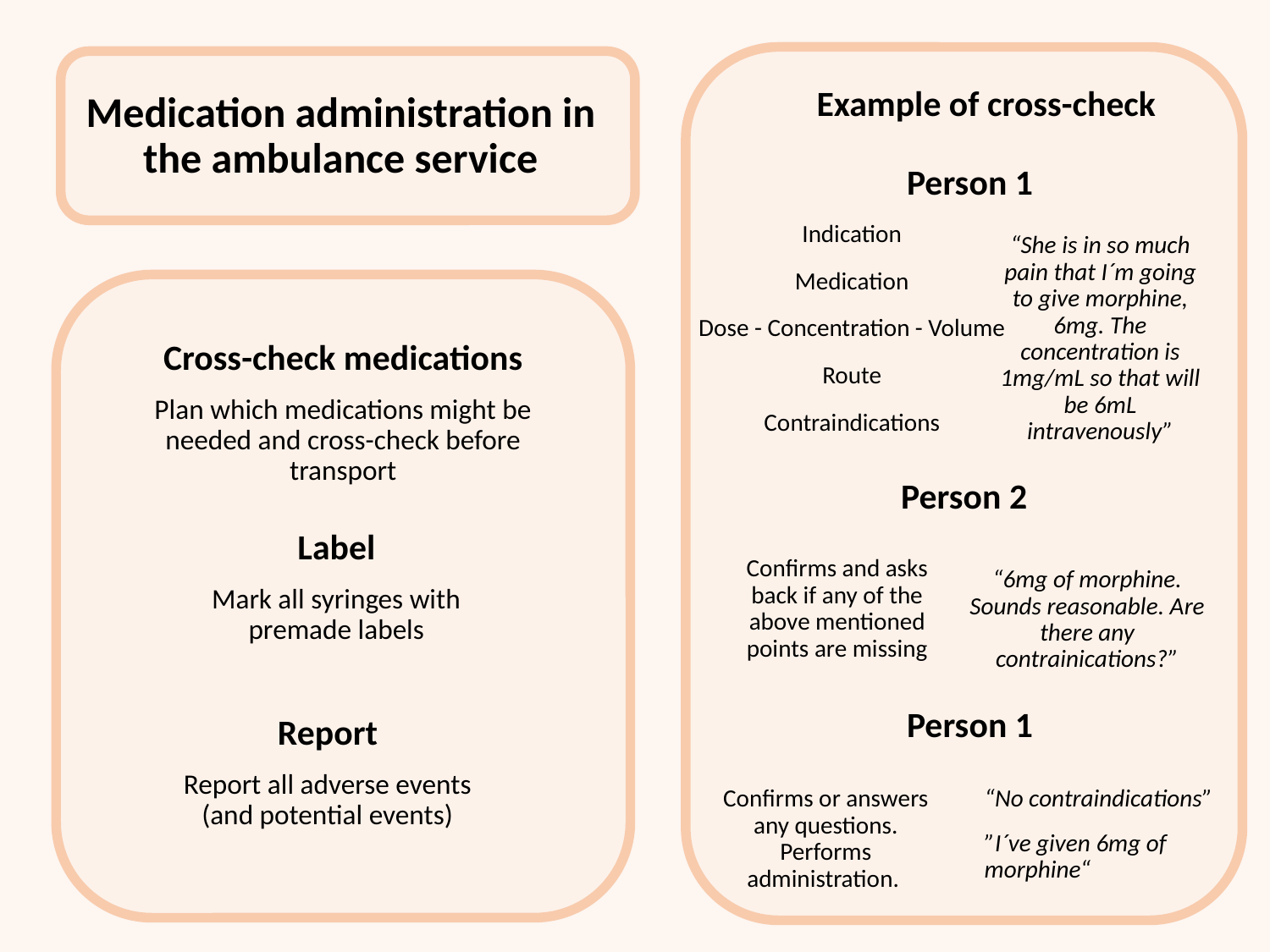

# Medication administration in the ambulance service
Example of cross-check
Person 1
Indication
Medication
Dose - Concentration - Volume
Route
Contraindications
“She is in so much pain that I´m going to give morphine, 6mg. The concentration is 1mg/mL so that will be 6mL intravenously”
Cross-check medications
Plan which medications might be needed and cross-check before transport
Person 2
Label
Mark all syringes with premade labels
Confirms and asks back if any of the above mentioned points are missing
“6mg of morphine. Sounds reasonable. Are there any contrainications?”
Person 1
Report
Report all adverse events (and potential events)
Confirms or answers any questions. Performs administration.
“No contraindications”
”I´ve given 6mg of morphine“
