# Supplementary material for: Medication administration errors in a Norwegian ambulance service: a quasi-experimental study on the impact of a team training program
Source: Scand J Trauma Resusc Emerg Med. 2026 Jan 24;34:41. doi: 10.1186/s13049-026-01560-1 (PMC12911085; doi:10.1186/s13049-026-01560-1)
Supplement: Supplementary file 3 — Additional file 3 [file 13049_2026_1560_MOESM3_ESM.docx]

**Team training intervention**

In this study, the intervention involved the implementation of the TeamSTEPPS team training program*, following three phases: phase 1—assessment and planning, phase 2—training and implementation, phase 3—sustainment. A selected group of ambulance professionals (APs) received master training to serve as TeamSTEPPS instructors and formed Change Teams together with the ambulance station leaders and the first author (KM). The Change Teams were responsible for planning the team training and selecting appropriate TeamSTEPPS tools and strategies to fit the needs of the ambulance service.

**Phase 1 - Assessment and planning**

The organization was assessed to be ready to undertake the intervention by the leadership of the prehospital division of the hospital trust. Central to TeamSTEPPS is the development of four key skills of teamwork (communication, leadership, situation monitoring and mutual support) and the use of tools to enhance those skills. The content and delivery of the TeamSTEPPS® intervention was tailored by the Change Teams towards the goals of the ambulance service (strengthen teamwork and improve medication administration and patient safety) and to facilitate implementation. Certain tools provided by TeamSTEPPS (ISBAR and briefing/debriefing checklists) were adapted by the Change Teams for ease of use and relevance and presented to the rest of the ambulance service for input and final revisions.

**Phase 2 - Training and implementation**

The introduction days

The intervention started with a one-day six-hour introduction made mandatory for all ambulance professionals who worked regular shifts at one of the seven ambulance stations. To allow for ongoing clinical duties, both groups had two introduction days to ensure that everyone could participate. In addition, an extra introduction day with similar content was held for those not able to attend the planned introduction days. The main objective of the introduction day was to establish the relevance of the intervention and the four key skills, and create motivation for change through lectures, group exercises and discussions.

The Change Teams spent one day preparing the content and delivery of the introduction day. Key TeamSTEPPS content was delivered by according to the TeamSTEPPS implementation guide with use of personal stories, group discussions and exercises to facilitate engangement among the participants.

The introduction days were held outside of their work environment to avoid disruptions and started with leaders of the ambulance service giving a short motivational speech of their vision for the intervention.

Training and implementation

Seven TeamSTEPPS tools, selected by the Change Teams and designed to strengthen the four key teamwork skills, were implemented during the four-month intervention period, see Figure 1. For each of the four months, the focus was on improving a single key skill (communication, team leadership, situation monitoring or mutual support) and the implementation of selected strategies and tools.

Figure 1 – Team training schedule


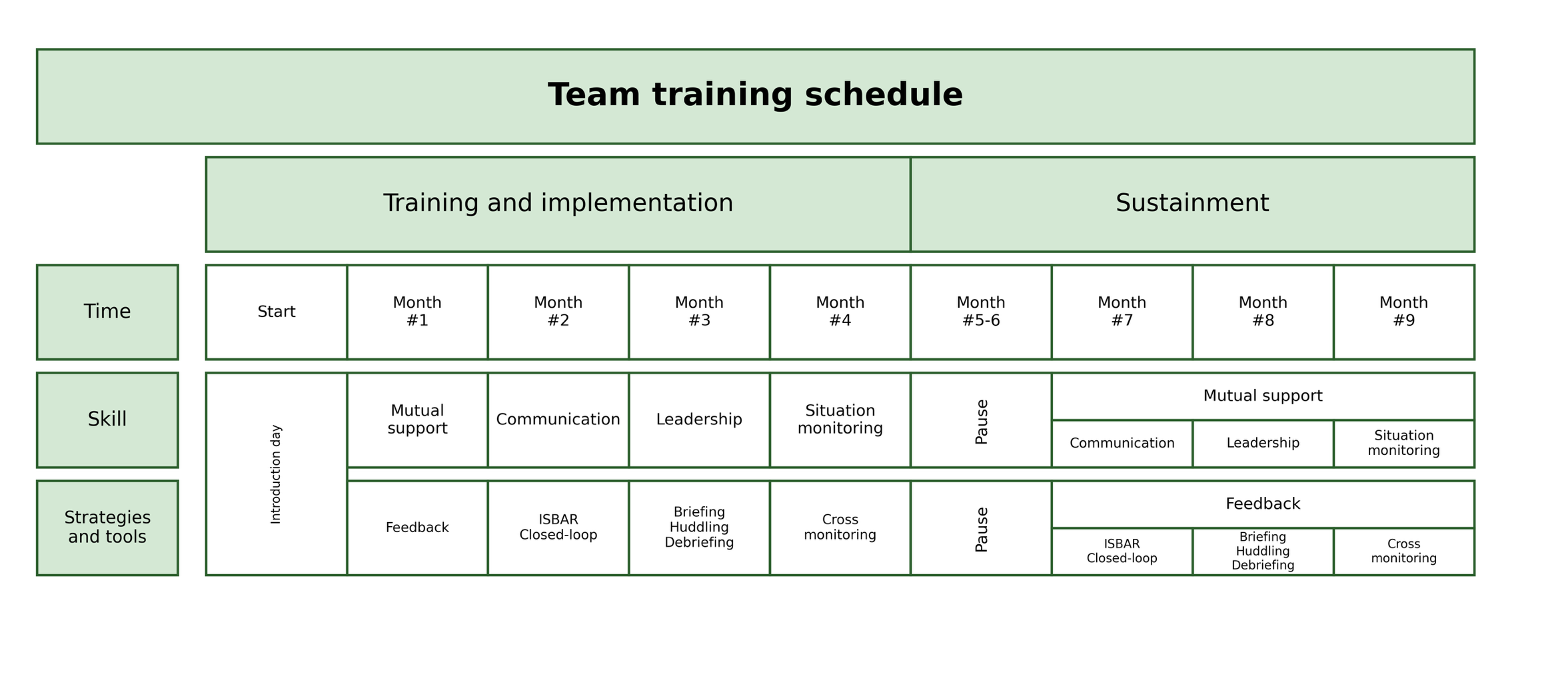


The skill of the month and selected tools were announced by unit leadership through an internal and app-based communications platform as well as morning meetings. The Change Team served as role models by using the tools and encouraging their colleagues to do the same. The unit leaders were motivated to remind the ambulance professionals about the study at every opportunity. Key skills and tools were also incorporated into regular training activities and credentialing by the division's medical instructor, for example using ISBAR in the telephone conversation with a hospital physician about a simulated stroke patient and giving feedback to their colleagues about the report after the simulation.

Particularly relevant to this study was the focus on situation monitoring during medication administration. The APs were encouraged to cross-check medications in a way inspired by the Medication Administration Cross Check procedure (see additional file 2), as well as to label all syringes and report adverse and potentially adverse events related to medication administration.

To display progress, the Change Teams created a "cork board" where use of the tools was highlighted by placing a pin when a tool was used and subsequently organized an ice cream stand (ISBAR in Norwegian) when a dedicated number of pins were placed (celebrate short-term wins to generate enthusiasm).

Midway through the four-month intervention period, a two-hour meeting was held among members of the Change Teams to evaluate the process so far and make necessary adjustments to the rest of the period.

**Phase 3 - Sustainment**

Following a two-month break after the intervention period, a three-month sustainment phase was conducted. The Change Teams and the primary author (KM) discussed how to create sustainability of the intervention. We decided to have three months of repetition where communication, leadership and situation monitoring would be in focus for a month each and to encourage ambulance professionals to provide verbal feedback to their colleagues (mutual support) on the use and development of teamwork skills throughout the period. Again, the skill of the month and the selected tools were announced by unit leadership using an app-based communications platform as well as staff briefings.

To foster sustainability beyond the intervention and research period, an effort was made to incorporate elements of TeamSTEPPS into already existing activities, thereby avoiding the need for additional resources. The division's medical instructor and unit leaders included, where relevant, selected key skills and implemented tools into scheduled training activities, evaluations, simulation training, and introduction of trainees and new employees.

Reference:

* Agency for Healthcare Research and Quality. TeamSTEPPS®. Rockville (MD): AHRQ; Available from: <https://www.ahrq.gov/teamstepps/index.html>. Accessed 13 Jan 2026.
