# Supplementary material for: Medication administration errors in a Norwegian ambulance service: a quasi-experimental study on the impact of a team training program
Source: Scand J Trauma Resusc Emerg Med. 2026 Jan 24;34:41. doi: 10.1186/s13049-026-01560-1 (PMC12911085; doi:10.1186/s13049-026-01560-1)
Supplement: Supplementary file 4 — Additional file 4 [file 13049_2026_1560_MOESM4_ESM.docx]

Additional file 4

Electronic patient journal review protocol

A. Pre-review process

Extraction

With permission from the cooperating hospital trust, electronic patient journals (EPJs) will be made available through the “access module”. Randomly selected EPJs from the predetermined time periods and following the criteria of inclusion and exclusion will be reviewed. The extracted data will be saved in a separated Excel file on a secure research server belonging to the hospital trust. All subsequent analysis will be done on the hospital trust secure research servers.

Number of journals extracted

The finite research on medication administration errors (MAEs) in the ambulance service is of varying methodology, has inconsistent definitions of what constitutes a MAE, and the frequency of MAEs reported vary substantially from 0.17% to 34.7%. Due to this variation in MAEs in the literature, we decided against performing a power calculation to estimate the number of EPJs needed to obtain an adequate sample size. Based on discussions with the project statistician, an initial 500 EPJs will be transformed into datasets and reviewed before an interim analysis will be performed. If required, more journals will be extracted and reviewed.

Inclusion criteria

- EPJs for patients who are transported or treated within the assigned areas
- Patient which receives medication, intravenously, by inhalation, intramuscularly, intraosseous, per os, sublingually, or intranasally. Examples of medications; morphine and esketamine. Oxygen and intravenous fluids such as Ringers Acetate and 0.9% Saline was not considered medication in this study.

Exclusion criteria

- Missing and/or incomplete data making the review process not possible

Data fields extracted

The mission characteristics in table 1 will be extracted automatically from the EPJ system. These data will be linked with the manually imputed data from the review process done in the access module.

Key map

All EPJ extracted from the hospital database have a unique personal identification number. A new randomly assigned unique ID will be generated and assigned to each EPJ, and a key will be made between the original unique personal identification number and the newly assigned unique ID. The new keymap will be stored at a secure server. This process ensures trackability, especially if incidental findings need to be tracked back to a patient.

Definitions

Patients who receive medications intravenously, intramuscularly, by inhalation, intraosseously, per os, sublingual or intranasally are defined as patients who have received medication. MAEs will be defined as deviations from the 5 R’s as defined by WHO and the standard operating procedures of the ambulance service and be subcategorized into:

- Wrong drug
- Wrong dose
- Wrong time
- Wrong route
- Other

Review process

Each extracted EPJs will be reviewed for MAEs. If any MAEs are found, they will be classified into one of the 5 R’s. The review team consists two candidates with experience from emergency medicine and the ambulance service, one paramedic and one physician. The reviewers will follow the review checklist (B) and carry out the process independently. If the independent reviews differ from each other, the electronic EPJs and classification are reviewed and discussed by both reviewers together. The review team will utilize a third expert reviewer if no classification can be agreed upon to reach consensus.

Unanticipated events

If the reviewers find examples of MAEs with the potential to have caused significant harm to patients, the reviewer will notify the project leader. The project leader will inform the project coordinator at the cooperating hospital trust. The coordinator at the hospital trust will evaluate the potential adverse event. The hospital trust can reidentify the patient using the keymap if the patient needs follow-up regarding the (possible) adverse events.

Interrater testing

To familiarize the reviewers with the review process, reviewers EG, BK, KM, and SS, will review ten random EPJs outside the study sample in an open panel using the review protocol. During this panel review, the necessary fields for analysis will be discussed and consensus will be sought in interpretation of the review protocol. The interrater reliability will be calculated amongst the reviewers for all included EPJs and be included in the study results.

B. Review checklist

An MAE is to be noted if deviations from the 5 R´s, as specified by the standard operating procedures (SOPs) of the ambulance service, is found. Deviations from protocol will not be noted as MAEs if explicit written explanation for said deviation is found in the EPJ and the decision is deemed appropriate by the reviewers. Nor if the care of the patient is by a physician with medication rights outside the scope of the ambulance service.

Wrong drug

An error is noted if:

- The drug is used outside of the indications defined in the SOPs.
- Patient presentation and/or vital signs contraindicates the use of the drug and if said contraindication is listed in the SOPs.
- There is a documented case of wrong drug given to a patient.

Wrong dose

An error is noted if:

- Deviation from dosage as described in the SOPs

In cases where dosage should be calculated by weight as defined by the SOPs, but no weight is documented, the case is not to be marked as an MAE (deviation from dosage). However, the EPJ is marked as “weight-dose omission”.

Wrong time

An error is noted if:

- The SOP dicatates a timing of medication(s) that are not followed.

Wrong route

An error is noted if:

- The administration route deviates from listed approved administration routes in the SOP

Other

An error is noted if:

The review team finds a medication administered outside the scope of the SOP but doesn’t fit in any of the other MAE subcategories.

Table 1 . Mission characteristics

| Variable name | Data type | Variable size | Example |
| --- | --- | --- | --- |
| Unique electronic patient journal ID from keymap | Number | 6 | 123456 |
| Study group 1 2 | Number | 1 | 1 |
| Weekday when arrival of ambulance at patient  1=Monday 2=Tuesday  3=Wednesday  4=Thursday  5=Friday  6=Saturday  7=Sunday  99= Missing data | Number | 1 | 1 (Monday) |
| Time of day of arrival of ambulance at patient HH:MM | Time | 5 | 14:02 |
| Time of day of arrival of ambulance at final destination with patient | Time | 5 | 14:02 |
| Medication name (to be linked in separate database, due to multiple medications/administrations) | Text | 100 | Paracetamol |
| Dosage (to be linked in separate database, due to multiple medications/administrations) | Text | 100 | 0,5mg |
| Administration route (to be linked in separate database, due to multiple medications/administrations) | Text | 100 | Rectal |
