# Supplementary material for: Medication administration errors in a Norwegian ambulance service: a quasi-experimental study on the impact of a team training program
Source: Scand J Trauma Resusc Emerg Med. 2026 Jan 24;34:41. doi: 10.1186/s13049-026-01560-1 (PMC12911085; doi:10.1186/s13049-026-01560-1)
Supplement: Supplementary file 5 — Additional file 5 [file 13049_2026_1560_MOESM5_ESM.docx]

# Full univariable and multivariable logistic regression analysis of medication administration errors

|  | **Univariable model** | | | | | | | **Multivariable model** | | | | | | |
| --- | --- | --- | --- | --- | --- | --- | --- | --- | --- | --- | --- | --- | --- | --- |
| **Variable** | **B** | **SE** | **Wald χ²** | **df** | **p-value** | **OR** | **95% CI** | **B** | **SE** | **Wald χ²** | **df** | **p-value** | **Adjusted OR** | **95% CI** |
| **Intervention** | 0.15 | 0.11 | 1.86 | 1 | 0.17 | 1.17 | 0.94-1.45 | 0.15 | 0.12 | 1.60 | 1 | 0.21 | 1.17 | 0.92-1.48 |
| **Group** | 0.08 | 0.11 | 0.50 | 1 | 0.48 | 1.08 | 0.87-1.35 | 0.06 | 0.13 | 0.23 | 1 | 0.63 | 1.06 | 0.83-1.36 |
| **Sex** | 0.07 | 0.11 | 0.42 | 1 | 0.52 | 1.08 | 0.86-1.34 | 0.09 | 0.12 | 0.56 | 1 | 0.46 | 1.10 | 0.86-1.39 |
| **Age** (ref: >69 years) |  |  | 2.21 | 5 | 0.82 |  |  |  |  | 3.12 | 5 | 0.68 |  |  |
| 0-5 years | -0.05 | 0.55 | 0.01 | 1 | 0.93 | 0.95 | 0.33-2.78 | 0.56 | 0.60 | 0.89 | 1 | 0.35 | 1.76 | 0.55-5.66 |
| 6-11 years | -0.07 | 0.61 | 0.01 | 1 | 0.91 | 0.93 | 0.28-3.06 | 0.12 | 0.63 | 0.04 | 1 | 0.85 | 1.13 | 0.33-3.85 |
| 12-17 years | 0.05 | 0.44 | 0.01 | 1 | 0.91 | 1.05 | 0.44-2.49 | -0.22 | 0.50 | 0.20 | 1 | 0.65 | 0.80 | 0.30-2.11 |
| 18-39 years | -0.18 | 0.18 | 0.97 | 1 | 0.32 | 0.84 | 0.59-1.19 | -0.13 | 0.20 | 0.42 | 1 | 0.52 | 0.88 | 0.60-1.30 |
| 40-69 years | -0.16 | 0.13 | 1.71 | 1 | 0.19 | 0.85 | 0.67-1.09 | -0.18 | 0.14 | 1.70 | 1 | 0.19 | 0.84 | 0.64-1.09 |
| **Mission start – weekday/weekend** | 0.05 | 0.13 | 0.14 | 1 | 0.71 | 1.05 | 0.82-1.34 | 0.13 | 0.14 | 0.86 | 1 | 0.35 | 1.14 | 0.87-1.50 |
| **Mission start –**  **day/night** | -0.13 | 0.12 | 1.25 | 1 | 0.26 | 0.88 | 0.70-1.10 | -0.14 | 0.13 | 1.19 | 1 | 0.28 | 0.87 | 0.68-1.12 |
| **Mission duration** | 0.00 | 0.00 | 1.18 | 1 | 0.28 | 1.00 | 1.00-1.01 | 0.00 | 0.00 | 0.13 | 1 | 0.72 | 1.00 | 1.00-1.01 |
| **Triage** (ref: Green) |  |  | 0.19 | 3 | 0.98 |  |  |  |  | 1.11 | 3 | 0.78 |  |  |
| Red | 0.02 | 0.21 | 0.01 | 1 | 0.93 | 1.02 | 0.68-1.53 | -0.05 | 0.24 | 0.04 | 1 | 0.84 | 0.95 | 0.59-1.53 |
| Orange | 0.00 | 0.20 | 0.00 | 1 | 0.99 | 1.00 | 0.68-1.47 | 0.03 | 0.23 | 0.01 | 1 | 0.91 | 1.03 | 0.66-1.60 |
| Yellow | -0.04 | 0.19 | 0.05 | 1 | 0.83 | 0.96 | 0.66-1.40 | 0.12 | 0.23 | 0.28 | 1 | 0.60 | 1.13 | 0.73-1.75 |
| **Medication doses administered**  (ref: 1 dose) |  |  | 21.22 | 2 | <0.01 |  |  |  |  | 1.28 | 2 | 0.53 |  |  |
| 2-3 Doses | 0.19 | 0.12 | 2.43 | 1 | 0.12 | 1.21 | 0.95-1.54 | -0.08 | 0.17 | 0.19 | 1 | 0.66 | 0.93 | 0.66-1.30 |
| 4+ Doses | 0.78 | 0.17 | 21.17 | 1 | <0.01 | 2.19 | 1.57-3.05 | 0.13 | 0.29 | 0.21 | 1 | 0.65 | 1.14 | 0.65-2.00 |
| **Number of different medications administered** | 0.35 | 0.06 | 29.43 | 1 | <0.01 | 1.42 | 1.25-1.61 | 0.38 | 0.11 | 11.91 | 1 | <0.01 | 1.47 | 1.18-1.82 |

B = unstandardized coefficient; SE = standard error; df = degrees of freedom; OR = odds ratio; CI = confidence interval; missions included in analysis, n = 1,333 (88.9% of 1,499 missions);

Overall classification accuracy: 69.1%
